# Integrated approaches to health: A paradigmatic, methodological and operational comparison of EcoHealth, One Health and Planetary Health

**DOI:** 10.1079/cabionehealth.2025.0029

**Published:** 2025-10-07

**Authors:** Jakob Zinsstag, Andrea Ford, Saana Jukola, Salome Bukachi, Chris Degeling, Maxine Whittaker, Hugo Mantila Meluk, Kristina Pelikan, Martin Röösli, Christina Zinsstag, Kathrin Heitz-Tokpa, Dominique Charron, Andrea Kaiser-Grolimund

**Affiliations:** 1 Swiss Tropical and Public Health Institute, Kreuzstr. 2, 4123, Allschwil, Switzerland; 2 University of Basel, Petersplatz 3, 4003, Basel, Switzerland; 3 Andrea Ford, University of Edinburgh, 5–7 Little France Road, Edinburgh, EH16 4UX, UK; 4 University of Twente, Drienerlolaan 5,7522, NB, Enschede, the Netherlands; 5 Institute of Anthropology, Gender and African Studies, University of Nairobi, P.O. Box 30197-00100, Nairobi, Kenya; 6 Department of Anthropology, Durham University, UK; 7 Australian Centre for Health Engagement Evidence and Values, University of Wollongong, 2522, NSW, Australia; 8 James Cook University, Bebegu Yamba Campus, 1 James Cook Drive, Townsville QLD, 4811, Australia; 9 Universidad del Quindío, Colección de Mamíferos, Centro de Estudios de Alta Montaña (CEAM), Programa de Biología, Facultad de Ciencias, Básicas, Universidad del Quindío, Armenia, Colombia; 10 Centre Suisse de Recherches Scientifiques en Côte d’Ivoire, Km 17 Route de Dabou, Adiopodoumé, 01 BP 1303, Abidjan, République de Côte d’Ivoire; 11 Dominique Charron, One Health Institute, University of Guelph, 50 Stone Rd East, Guelph, Ontario, N1G 2W1 Canada

**Keywords:** One Health, EcoHealth, Planetary Health, public health, environmental health, natural foci of diseases, medical anthropology, veterinary public health, ontology, epistemology

## Abstract

While clinical medicine essentially concentrates on the human body and its parts, public health focuses on the health of human populations and their social and environmental determinants. Integrated approaches to health extend the focus of attention to humans in their socio-cultural and ecological environment and their mutual interdependencies, paying attention to inter-species interdependencies. Since the beginning of the 21st century, ecosystem approaches to health (EcoHealth), One Health and Planetary Health have emerged as integrated approaches that relate to and expand public health and related fields. In this article, we aim at clarifying their respective definitions, philosophical foundations and methodological positions. This clarification is important because the way we define integrated approaches to health shapes research, teaching methods and their translation into policy and practice. Key methods and case studies are summarized and compared. Among the three integrated approaches, EcoHealth and Planetary Health operate largely in academic networks and non-governmental organizations (NGOs). One Health is currently operationalized at the level of international organizations, regional organizations and national governments. Integrated approaches to health require urgent adoption and implementation, as they are pivotal for complex problem-solving regarding challenges such as pandemic prevention, climate change, biodiversity loss and antimicrobial resistance.

## Introduction

In this article, we compare the different paradigms of EcoHealth, One Health and Planetary Health as integrated approaches to health. We examine how the three concepts relate to or expand the more established disciplines of public health, environmental health and medical anthropology. Earlier analyses addressed the convergence of EcoHealth and One Health (Zinsstag, 2012; Harrison *et al*., 2019), the differences of values of EcoHealth, One Health and Planetary Health (McMichael, 2013; Lerner and Berg, 2017; Assmuth *et al*., 2020; Winkler *et al*., 2025) and a comparison of One Health and Planetary Health (de Castaneda *et al*., 2023). In recent years, some of the concepts came into the focus of attention with complex problems like the COVID-19 pandemic, climate change, biodiversity loss, antimicrobial resistance, civil unrest, open war and mass migration. Concurrently, international organizations of the United Nations, state organizations like the G7, G20, the Economic Community of West African States (ECOWAS) or the European Union, many national governments and NGOs engaged with One Health in a way that warrants a re-examination in a highly dynamic context of rapidly changing paradigms, positions, methods and policy.

### Methodological Considerations

The authors represent the fields of (social/medical) anthropology, philosophy, ethics, social sciences, biology, ecology, environmental health, wildlife conservation, veterinary epidemiology and public health. Most of them are involved in one or several integrated approaches to health and, through their work, have encountered different/various understandings and means of implementation of each of these approaches. In this article, we do not aim at an exhaustive literature review, but the authors contribute landmark references for their respective fields as part of an iterative consensus-finding process on theoretical foundations, methodological approaches and the level of operationalization between the different authors. The consensus finding process took place through direct discussions between authors, including additional authors with competencies in specific fields and by several rounds of circulating manuscript versions.

### Public Health and Related Fields

While clinical medicine concentrates on the individual human body, public health addresses the health of human populations. Related disciplines include epidemiology, social medicine, health promotion and prevention, health services research, health monitoring, health economics, health policy and health/medical ethics. A commonly used definition is: “The art and science of preventing disease, prolonging life and promoting health through the organized efforts of society” (Acheson, 1988), although many argue for a broadening of this definition to include the societal, cultural, commercial, ecological and economic determinants of health (Azari and Borisch, 2023). Interdisciplinary links from public health to other fields of knowledge have emerged. In this article, we highlight three of them that we consider relevant for the further development of integrated approaches to health: environmental health, veterinary public health, and medical anthropology.

**Fig. 1:**
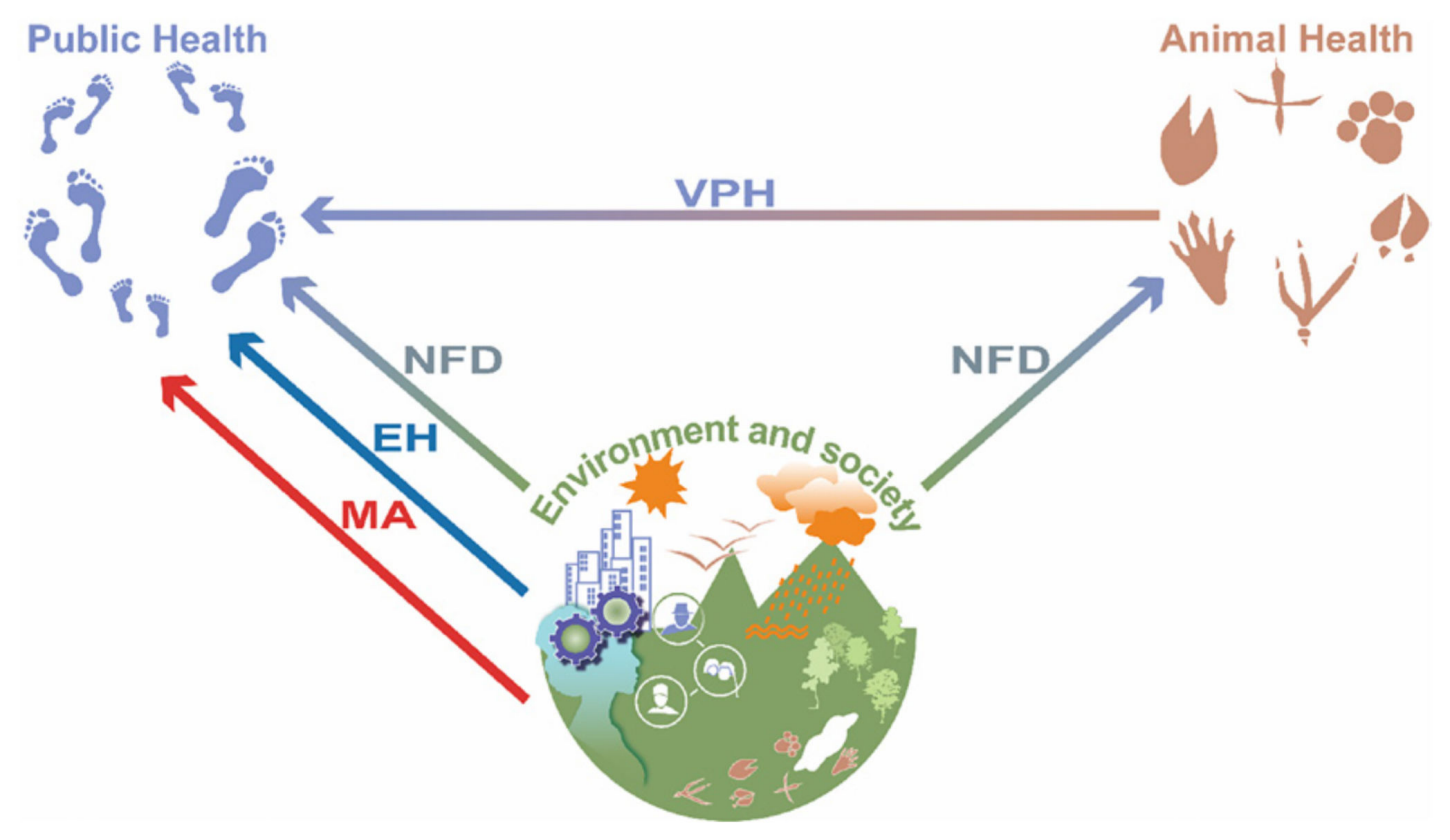
Graphical representation of various disciplines in an integrated framework: EH = Environmental health, NFD = Natural foci of infectious diseases, VPH = Veterinary Public Health and MA = Medical Anthropology.

- “*Environmental Health*” (EH) and related ideas emerged in the mid-19th century (Santos *et al*., 2019; Malsch *et al*., 2024). A classic example that inspired EH is how John Snow addressed the cholera epidemic in London in 1854 by systematically mapping patients to identify the source of contaminated water they used. EH focuses on how environmental factors impact human health (Fig. 1). This involves the effects of air quality, water and sanitation, chemical exposure, climate change, noise, radiation and other chemical and/or physical exposures. Environmental health initiatives have led to massive improvements in public health through laws on clean air and water, sanitation, chemical contamination and noise reduction. However, in many countries, there is still no ubiquitous access to clean drinking water, no environmental sanitation, pressing air and noise pollution and chemical contamination. The Russian zoologist Yevgeni Pavlovsky (1884–1965) advanced two important concepts in EH: “natural foci of diseases” (NFD, Fig. 1) and “landscape epidemiology”, identifying pathogens causing human and animal diseases from wildlife sources. For example, in Mongolia, marmots carry the causative agent of human plague, *Yersinia pestis*, without apparent illness. He founded the study of natural reservoirs of infectious disease in humans and animals, known as disease ecology. This concept has become very influential with increasing pandemic risks from wildlife. Earlier proponents were, among others, Carlos Juan Finlay (1833–1915), a Cuban physician and scientist renowned for his groundbreaking research on yellow fever, identifying *Aedes aegypti* as vector, or Joseph Grinnell (1877–1939), an influential American ecologist and biologist, best known for his pioneering work in ecology, biogeography, and conservation, laying foundational ideas that connect to the modern “One Health” concept.
- *“Veterinary Public Health”* (VPH), was originally conceived by James H. Steele (1913–2013) at the Centers for Disease Control (CDC) in Atlanta, USA after 1945. It is defined as the contribution of veterinary medicine to public health (Fig. 1). VPH addresses diagnosis, surveillance, and control of zoonotic diseases, food safety, laboratory animal medicine, biomedical research, biologics and pharmaceuticals. It is well established in international organizations, governmental administrations and academia (Steele, 1949).
- “*Medical Anthropology*” (MA) emerged in the middle of the 20th century as a distinct subfield of socio-cultural anthropology, studying the ways individuals and human systems cause, respond to, and make sense of health, disease, and illness (Baer *et al*., 2003). In 1972, the Society for Medical Anthropology (SMA) became a formal section of the American Anthropological Association, with similar initiatives in Britain and other countries following soon after (Coreil, 2008). Medical Anthropology’s contribution to understanding the social and cultural components of health and health care practices emphasizes socio-economic and global inequalities and historic injustices, thereby contextualizing “the social” quite broadly in time and space and recognizing how much “the environment” is shaped by human activity and vice-versa. Critical medical anthropology, in particular, emphasizes how ecological aspects and interventions shape health and wellbeing (Brown and Nading, 2019). Concepts in the field describe both a multiplicity of perspectives on disease (e.g. cultural epidemiology, Weiss, 2001) and the multiple and compounding causes of ill health (e.g. ‘syndemics’ (Singer and Clair, 2003)). In recent years, “more-than-human” health has emerged as a new area of interest in medical anthropology, concerned with how multispecies wellbeing is affected in positive and negative ways through human-animal relations (Kirksey and Helmreich 2010; Haraway, 2016; Brown and Nading, 2019).

Integrated approaches to health emerged in the 20th and 21st centuries (Fig. 1). They engage in interdisciplinary collaboration between medicine, public health, veterinary science, ecology, and social sciences and embrace complexity (Zinsstag *et al*., 2012), process philosophical (Barbour, 1997) and systemic thinking (Rüegg *et al*., 2018) at multiple levels of influence between science and society.

## Methods

### Integrated Approaches To Health

We acknowledge the holistic knowledge and belief systems of many indigenous groups around the world, that link human wellbeing with that of animals and the natural world around them. Moreover, the metaphorical extrapolation of the universe and/or nature as a living entity capable of illness or healing represents one of the earliest integrated visions of health (Lovelock, 1972; Johnston *et al*. 2013). In contrast, Western science from the period of Enlightenment onwards embraced dualistic paradigms that separated humans from the environment, and mind from matter, which led to progress through reductionist approaches and consequently a loss of integrated thinking. Highly specialist disciplinary sciences, in turn, are unable to tackle complex systemic problems like climate change or biodiversity loss. We acknowledge that there is a more complex variety of approaches within, among, and between indigenous and scientific knowledge traditions (Hillier *et al*., 2021), yet we emphasize the general principle that a change of paradigm is needed toward integrated approaches and a close inter- and transdisciplinary cooperation in science (Nowak and Coakley, 2013; Pelluchon, 2021; Gabriel, 2022). For the period before the 20th century, we refer the reader who is interested in the history of integrated approaches to health to “Animals and the shaping of modern medicine: One Health and its histories” (Woods *et al*., 2017). In this article, we limit our reflection to the 20th and 21st centuries.

### “Ecosystem Approaches To Health” (Ecohealth)

**Fig. 2:**
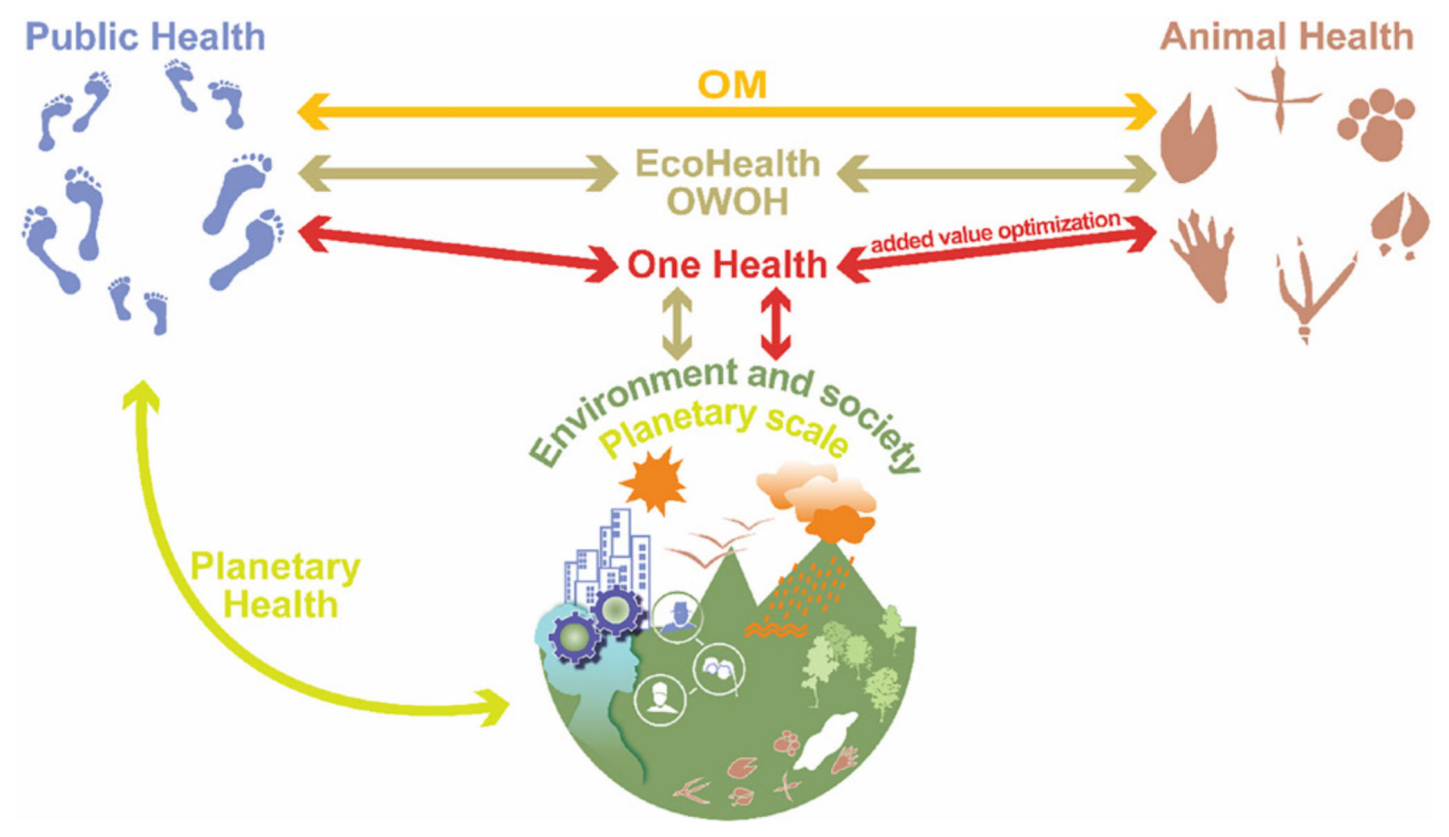
Graphical representation of the integrated approaches to health. OM = One Medicine, EcoHealth = Ecosystem approaches to health, OWOH = One World One Health™, OH = One Health, PH = Planetary Health.

**Fig. 3:**
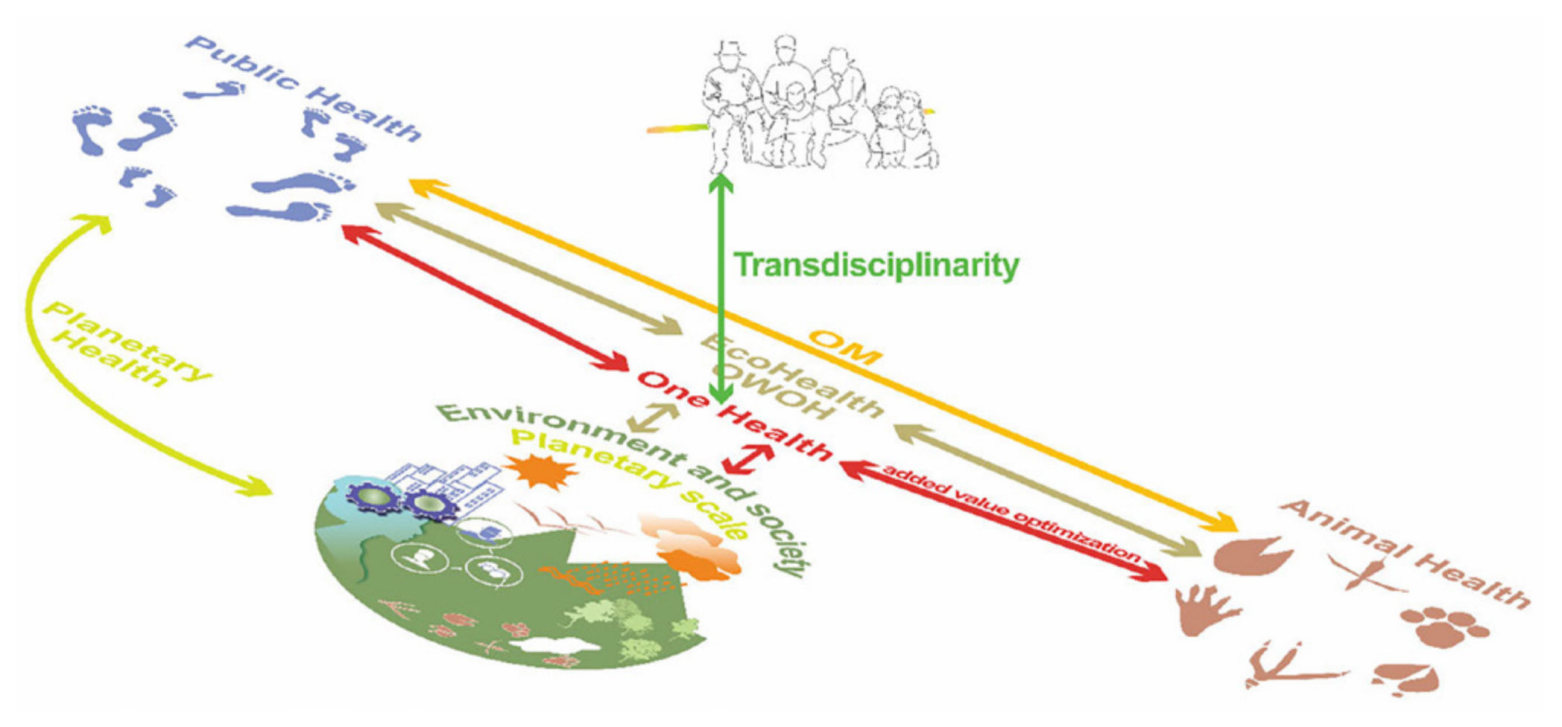
Graphical representation of the paradigmatic comparison of integrated approaches to health. OM = One Medicine, EcoHealth = Ecosystem approaches to health, OWOH = One World, One Health, Planetary health and transdisciplinarity.

Drawing on ecosystem management concepts that were successfully used in the 1980s and 1990s to remediate pollution and improve fisheries and water quality in the Laurentian Great Lakes (Guthrie *et al*., 2019), scholars developed “Ecosystem Approaches to Health” (EcoHealth). This approach applied emerging ecological and complex systems theories to address interconnected population, animal and environmental health problems (Waltner-Toews, 2001). In the same period, scholars explored the concept of ecosystem health, encompassing the health of humans, animals and other elements of ecosystems (Rapport *et al*., 1998, 1999). EcoHealth is defined as a systemic approach to understanding dynamic mutually influencing processes between humans, animals and ecosystems. The framework is based on a concept of nested socio-ecological systems and addresses gender and social equity and justice (Fig. 2). Transdisciplinary processes are employed that couple academic science, participatory action research, and practical knowledge in the co-production of transformational knowledge (Charron, 2012a, 2012b) (Fig. 3). Scholars studying mercury contamination of fish in theAmazon and subsequent human exposure to mercury contributed conceptual frameworks and methods for Ecosystem Approaches to Health. The Canada-Brazil project innovatively brought environmental toxicologists and physiologists, physical geographers, biogeochemists, anthropologists and others to work together in the field with communities. A key finding was that fish contamination with mercury was not primarily due to pollution from upstream goldmining as documented in other areas, but rather to the washing out of naturally occurring mercury from the soil exposed by slash and burn deforestation. Reforestation showed a reduction of mercury contamination in fish and humans (Forget and Lebel, 2001; Guimarães and Mergler, 2012). This transdisciplinary endeavor led the team to new insights, and influenced foundational concepts for Ecosystem Approaches to Health at the International Development Research Centre, part of Canada’s official development assistance, that funded the mercury study in Brazil and supported capacities, methods and research for EcoHealth, globally and in Canada (Webb *et al*., 2010, 2023).

In their ratified constitution, the International Association for Ecology and Health defined EcoHealth as follows: “Ecohealth, strives for sustainable health of people, animals, and ecosystems by promoting discovery and understanding through transdisciplinary action-research. It encourages problem solving that draws upon multiple types of knowledge from the natural, social and health sciences, and the humanities” (IAEH ratified constitution 2008, cited in (Zinsstag, 2012). Communities of practice in universities in dozens of countries, initially supported by the International Development Research Centre (IDRC) and other Canadian agencies in the 2000s as capacity building efforts, became self-sustaining in Canada and many regions (Webb *et al*., 2023), joining and contributing to the expansion and development of One Health.

### From “One Medicine” To “One Health”

The American veterinary epidemiologist Calvin Schwabe, while at the American University in Beirut, studied the health of Dinka pastoralists and their animals in South Sudan. The way of life of Dinka pastoralists closely linked with their animals inspired him to coin the term “One Medicine” (OM), meaning that “there is no difference of paradigm between human and veterinary medicine. Both sciences share a common body of knowledge in anatomy, physiology, pathology, on the origins of diseases in all species.” (Schwabe, 1984). One Medicine is the first integrated concept that considers the mutual interdependence between human and animal health and, consequently, promotes a close connection between human and veterinary medicine, caring for the health of animals as much as for people (arrows going in both directions in Fig. 2).

In the wake of the first SARS Coronavirus global outbreak in 2003, veterinary leaders at the Wildlife Conservation Society (WCS) used the term “One Health” and emphasized “the essential link between human, domestic animal and wildlife health and the threat disease poses to people, their food supplies and economies, and the biodiversity essential to maintaining the healthy environments and functioning ecosystems we all require” (Cook *et al*., 2004; Pettan-Brewer *et al*., 2024). In 2003, WCS also launched the AHEAD (Animal Health for the Environment And Development – later to become Animal and Human Health for the Environment And Development) program at the World Parks Congress in Durban, South Africa (Osofsky *et al*., 2005). When explicitly addressing links between oceans, wildlife, and human health, Wilcox and Aguirre, having previously mostly engaged in EcoHealth, used the term “One Ocean, One Health” (Wilcox and Aquirre, 2004). They are quoted as having used the term “One Health” for the first time in biomedical literature (Pettan-Brewer *et al*., 2024).

In 2004, WCS issued “The Manhattan principles on One World, One Health™” (OWOH), establishing a One Health paradigm based on the idea that: “The earnestness and effectiveness of humankind’s environmental stewardship and our future health have never been more clearly linked. To win the disease battles of the 21st Century while ensuring the biological integrity of the Earth for future generations requires interdisciplinary and cross-sectoral approaches to disease prevention, surveillance, monitoring, control and mitigation as well as to environmental conservation more broadly” (Cook *et al*., 2004; Osofsky *et al*., 2005).

In 2005, Zinsstag and co-workers introduced the extended “One Health” concept in human and animal health systems with a paradigmatic extension, requiring to demonstrate an added value resulting from a closer cooperation between human and animal health and other involved sectors as a first theoretical pillar (Zinsstag *et al*., 2005). The paradigm of added value was validated by demonstrating the benefits of joint human and animal health services for mobile pastoralists in Chad (Schelling *et al*., 2007a), the societal benefits of livestock brucellosis vaccination for public health in Mongolia (Roth *et al*., 2003), and the advantage of dog mass vaccination to eliminate human rabies (Zinsstag *et al*., 2009) (Fig. 2).

One Health High Level Expert Panel (OHHLEP) was convened in early 2021 by the quadripartite agencies (WHO, FAO, WOAH and UNEP) at the request of the UN Secretary General in response to the COVID-19 pandemic and the need to urgently understand the emergence and spread of zoonotic pandemics. In 2022, the OHHLEP, advising the quadripartite international organizations published a new definition of One Health as “an integrated, unifying approach that aims to sustainably balance and optimize the health of people, animals and ecosystems. It recognizes that the health of humans, domestic and wild animals, plants, and the wider environment (including ecosystems) are closely linked and inter-dependent” (One Health High-Level Expert *et al*., 2022). This definition extends the earlier definition of “added value” toward the *optimization* of the health of humans, animals, plants and the environment (Fig. 2). It opens a range of interpretative questions on the normative relationship between humans, animals and the environment, and it has wide ranging philosophical, institutional and governance consequences (SAPEA, 2024).

### “Planetary Health”

The groundwork for Planetary Health was established by the Health and Ecosystems: Analysis of Linkages (HEAL) consortium in 2013, with their “Human Health Impacts of Ecosystem Alteration”, which emphasized: “Human activity is rapidly transforming most of Earth’s natural systems. How this transformation is impacting human health, whose health is at greatest risk, and the magnitude of the associated disease burden are relatively new subjects within the field of environmental health. It addresses human health implications of changes in the structure and function of natural systems and how these changes are affecting human health. Such efforts could lead to a more robust understanding of the impact on human health, accelerating environmental change and inform decision making in the land-use planning, environmental conservation, and public health policy realms (Myers *et al*., 2013). In 2014, Judith Rodin proposed to call the new concept “Planetary Health”, which led to the “Safeguarding Human Health in the Anthropocene Epoch: Report of The Rockefeller Foundation–*Lancet* Commission on Planetary Health,” (Whitmee *et al*., 2015), wherein Planetary Health was defined at a high level as “the achievement of the highest attainable standard of health, wellbeing, and equity worldwide through judicious attention to the human systems—political, economic, and social—that shape the future of humanity and the Earth’s natural systems that define the safe environmental limits within which humanity can flourish. Put simply, planetary health is the health of human civilization and the state of the natural systems on which it depends”.

Operationally, Planetary Health was subsequently more clearly defined as “a field focused on improving the understanding of, and ability to measure, the public health impacts of anthropogenic environmental change, so as to inform decision-making in the land-use planning, ocean-use planning, environmental conservation, and public health policy realms”, relating to the HEAL consortium’s earlier 2013 analysis. Osofsky and Pongsiri (Osofsky and Pongsiri, 2018) also reminded us that “what cannot be measured cannot be managed.” Planetary Health is thus an extension of Environmental health to the planetary level, acknowledging that the state of the planet affects and interacts with ecosystems, including animal and public health. Practically, it is still focused on human health (McMichael, 2013) (Fig. 2), but conceived of with a drive to better define and articulate the co-benefits of environmental stewardship. In that sense, health was being envisioned as a derivative ecosystem service (Osofsky pers. Comm).

### The Use Of Transdiscipilinarity In Integrated Approaches To Health

A further theoretical pillar of all three integrated approaches to health, EcoHealth, One Health, and to lesser extent Planetary health, is their transdisciplinary approach (Available at: www.transdisciplinarity.ch, accessed 10 July 2025), actively involving non-academic actors from communities, various levels of authorities, the private sector and non-governmental organizations in the research and the research-to-action process as project partners (Charron, 2012b). Transdisciplinarity works on/entails the understanding that non-scientific actors must be regarded and included as experts in their specific circumstances, generating and operating on their own form of practical knowledge. Integrated approaches to health are thus simultaneously “*interdisciplinary*”, meaning that they work in-between academic disciplines like medicine and sociology; and “*transdisciplinary*” oriented, meaning that they work between academic and practical ways of knowledge generation, depending on the phase of research (see Fig. 2 (Herweg *et al*., 2012)). In the early 2000s, the development of One Health at the University of Basel and the Swiss TPH was strongly influenced by anthropological and sociological concepts of “access to care” and “multilayered social resilience” (Obrist *et al*., 2007, 2010), which led to mixed qualitative-quantitative methods for understanding “equity effectiveness” (Zinsstag *et al*., 2006, 2011). The role of social sciences in One Health was reflected already in 2009 when bringing “syndemics”, multispecies ethnography, food anthropologies, economics and indigenous knowledge into One Health discussions (Rock *et al*., 2009; Whittaker, 2015). One Health, thus, attempts to be epistemologically open to other ways of knowledge generation for societal problem solving in research/research application partnerships (Schelling *et al*., 2007b; Zinsstag *et al*., 2023b) (Fig. 3). One Health follows a similar approach to EcoHealth, valuing indigenous and local knowledge and engaging with the humanities to introduce reflective thinking and critical analysis (Charron, 2012b). Figure 3 depicts the interdisciplinary academic bottom plane with the orthogonal transdisciplinary interaction to emphasize the aimed-at multi-epistemic nature of integrated approaches to health.

## Results

### Comparison Of Paradigms, Philosophical Foundations and Methods Of Integrated Approaches To Health

Integrated approaches to health combine multiple disciplinary perspectives on the human-animal-environment-nexus, extending the focus of attention from humans only (anthropocentric perspective) to humans in their socio-cultural and ecological environment, and to ecosystems. In this section, we compare the above-mentioned EcoHealth, One Health and Planetary Health approaches for their philosophical foundations. We examine their underlying paradigms, their worldviews, as well as ontological, epistemological and methodological assumptions. This clarification is important for the potential of their convergence and because the way we define integrated approaches to health shapes their methods and the stream of research and its translation into policy and practice.

Importantly, we note that each approach has a different political orientation and agenda: EcoHealth emerged from communities working on similar concepts from both international public health and human rights, as well as conservation perspectives. One Health was developed by disease ecologists (Wilcox and Aquirre, 2004), conservationists (Cook *et al*., 2004), and veterinarians, while Planetary health was conceived by medical doctors and climate scientists. In each case, the approach and vision are shaped by the scientific interest and political ambitions of these distinct communities. While this acknowledgement is important for an understanding of the way the approaches are and have been enacted, here our concern is with the conceptual underpinnings of each. We intentionally develop a phenomenological approach that sets aside these political dimensions.

EcoHealth and One Health are aligned in that their core values include humans, animals, plants and ecosystems, whereas Planetary Health focuses exclusively on individual and population health of humans (Lerner and Berg, 2017), being anthropocentric by design (Almada *et al*., 2017). EcoHealth and One Health also emphasize the need for transdisciplinary work. Paradigmatic differences between “environment” and “ecosystem” as ways of conceptualizing the non-human are instructive here. As defined in the One Health SAPEA report (SAPEA, 2024), an ecosystem is a holistic system comprised of living and non-living entities that are both interconnected and interdependent, while the term environment implies that a person (or sometimes an animal or a species) is the “figure” on an environmental “background” or surrounded by an environmental “context” – meaning that the environment is a *place* instead of a collective of entities and processes in which humans are implicated. By contrast, humans and animals are *part of* ecosystems. Ecosystems are plural and localized, but also interconnected with and interdependent on each other such that the planet can also be described as an ecosystem (Holling, 2001). Both EcoHealth and One Health engage with the concept of ecosystems, re-integrating humans into a matrix of dependencies and prerogatives, though not uniformly. Planetary Health, insofar as it has been anthropocentric, is the least “ecosystemic” of the three. Euro-American philosophical and scientific history has emphasized human exceptionality and separation instead of integration, making it difficult to move beyond the concept of an environment external to humans and full of “resources” for human use. All three approaches are affected by this limitation. They call for a radical paradigm shift and a commitment that may not be available as of yet, as it requires action given the present organization of social and governmental systems (Kickbusch and Bright, 2024).

### Epistemological Positions and Integrated Methods

What reality is taken to be (ontology) and the way we generate the knowledge to describe that reality (epistemology) are central elements of a philosophy of science. An analysis of EcoHealth and One Health helps to reveal similarities and differences in their philosophical assumptions. Both are collaborative, interdisciplinary and systems focused approaches at the human, animal and ecosystem health interface. However, the first leans toward constructivist assumptions and the latter more toward positivism with overlaps at the conceptual level (Harrison *et al*., 2019). Some fundamental epistemological assumptions are related to the way in which the role of different experts is perceived. For example, most One Health approaches clearly incorporate inter- and transdisciplinary engagement between academic experts and societal actors and hence become also more constructivist (Whittaker *et al*., 2020; Zinsstag *et al*., 2023b). Planetary Health’s focus on the health effects of the earth’s natural systems can be considered as largely positivist (Johnson *et al*., 2024), including more and more also a constructivist stance (Hoogeveen *et al*., 2023).

Openings to epistemic diversity emerge whenever actors within any of these three approaches engage in transdisciplinary processes, including incorporating local and indigenous knowledge (Charron, 2012b; Zinsstag *et al*., 2022, 2023b). Epistemic diversity clearly also emerges in interdisciplinary approaches (Moon and Blackman, 2014). While EcoHealth very early-on considered health and environmental equity as a socio-political orientation (Bunch *et al*., 2011; Charron, 2012b), early One Health economic case studies referred to a liberal capitalist position (Roth *et al*., 2003) with a transition to social and environmental equity when applying an “equity-effectiveness” framework (Zinsstag *et al*., 2011a). A recent discussion paper advocates that Planetary Health engages in health equity, environmental justice and an economy of well-being that prioritizes health and well-being over traditional growth metrics (Brady, 2023). The “doughnut economy” framework for sustainable development combines the concept of planetary boundaries with the complementary concept of social boundaries (Available at: www.instituteofsustainabilitystudies.com, accessed 10 July 2025).

Different ontological and epistemological assumptions are reflected in the methods used in the approaches. EcoHealth methods emphasize systems thinking recognizing the interconnectedness of ecosystems, human health and societal factors. Transformational knowledge is co-produced from participatory transdisciplinary processes between researchers and communities and authorities. Special attention is given to gender and social equity, as well as ecological sustainability. EcoHealth is strongly action oriented, aiming at translating research findings into practical interventions and policy changes (Charron, 2012a, 2012b; Parkes, 2020). Planetary Health similarly applies systemic and transdisciplinary approaches with a clear focus on human health within the context of earth’s natural systems (Lerner and Berg, 2017). Health Impact Assessments (HIA) are recommended as a key method for Planetary Health operationalization (Osofsky and Pongsiri, 2018). One Health methods largely subscribe to the above approaches, while some aim at demonstrating an incremental benefit from the cooperation between different sectors in terms of saved lives of humans and animals, financial savings, improved resilience and sustained ecosystem services (Zinsstag *et al*., 2020).

With the advent of OHHLEP’s One Health definition as an “integrated, unifying approach that aims to sustainably balance and optimise the health of people animals and ecosystems” (One Health High-Level Expert *et al*., 2022), integrated approaches to health are challenged methodologically to bring together the health of humans and animals, plants and other species with the management of natural resources. The definition’s emphasis on “mobilizing multiple sectors, disciplines and communities at varying levels of society to work together to foster well-being and tackle threats to health and ecosystems, while addressing the collective need for clean water, energy and air, safe and nutritious food, taking action on climate change, and contributing to sustainable development”, poses myriad political, ethical and methodological challenges. Stronger methodologies are needed for conducting One Health research and implementation.

**Fig. 4:**
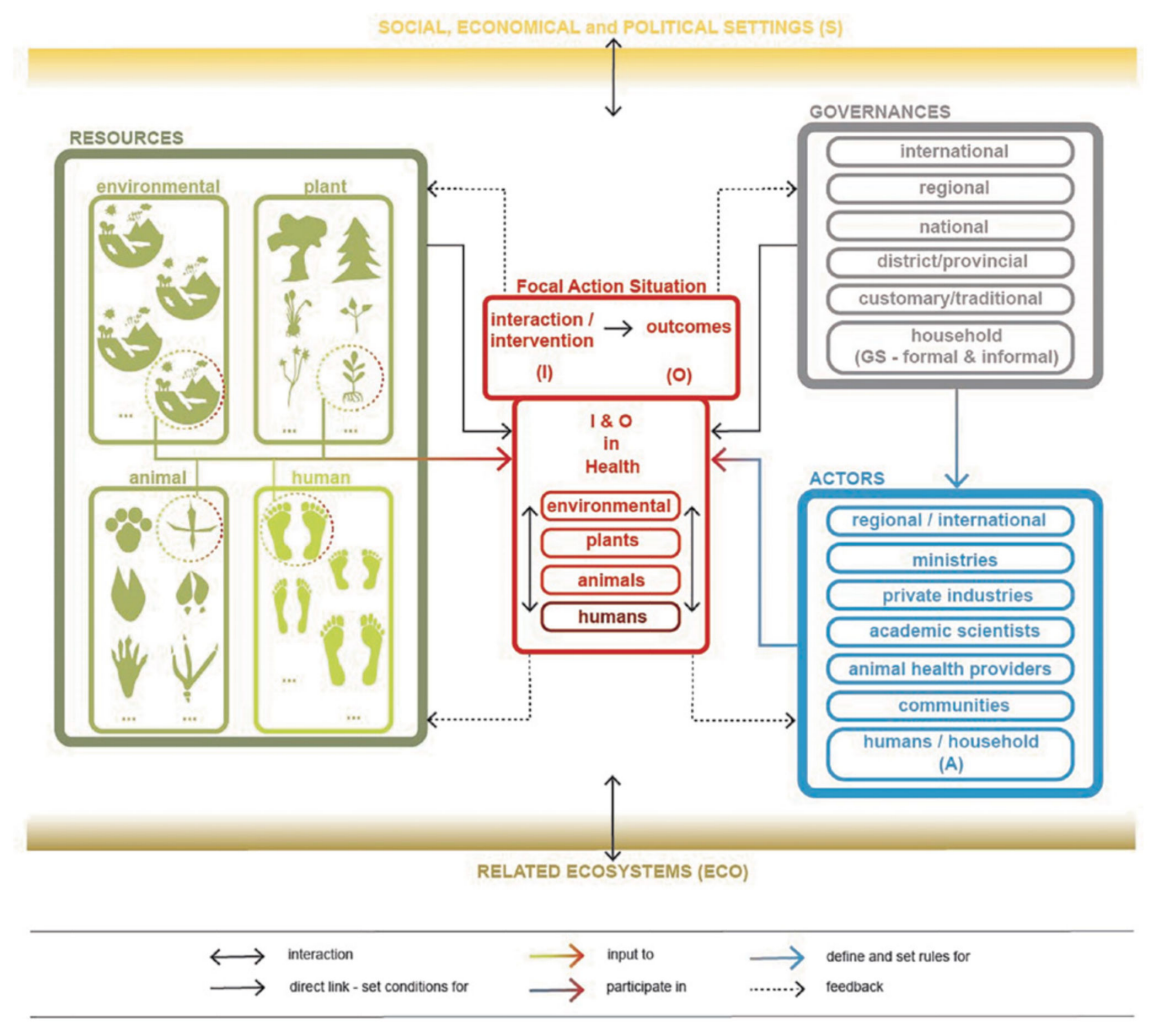
One Health in Social – Ecological Systems framework expanding natural resources with humans including their diversity as a resource and a scaled set of actors from household to nation (Zinsstag *et al*., 2024).

Drawing on the work of the Noble Prize winning economist Elinor Ostrom (Ostrom, 2015), we explore how game theory provides one option for rigorously modeling complex One Health problems. Ostrom was responding to the theories about the challenges in equitably sharing and governing global commons, such as land, water and air. This “tragedy of the commons”, coined by Hardin, assumes that commonly pooled resources are inevitably overused and depleted in the absence of external control (Hardin, 1968). Such concepts gained traction in the sustainable development institutions and policies that followed from the 1987 Brundtland report Our Common Future (World Commission on the Environment and Development, 1987) and the Rio de Janeiro Sustainable Development Summit in 1992. She argued instead that communities can create their own governing systems to avoid this tragedy. Ostrom proposed a social-ecological systems (SES) approach composed of resource systems, resource units, actors and governance systems (McGinnis and Ostrom, 2014). The SES approach by Ostrom and co-workers has been expanded to include humans as resources in the resource systems and resource units. Humans contribute to the national income by their per-capita contribution to the gross domestic product as human capital benefit. Illness or premature death leads to a loss of human capital benefit. In this way, human health can be formally included in the Ostromian SES. Animal health can be included similarly in the existing SES as a reduction of natural resource units from illness or premature death. Ostrom’s SES is thus extended to One Health in Social-Ecological Systems (OHSES) within a stringent mathematical and economic game theoretical framework (Fig. 4) (Zinsstag *et al*., 2024). Essentially, the framework considers actors that make choices about their strategies that affect the focal action process based on governance systems at different scales. In this way, the OHSES framework combines the sustainable management of natural resources and the health of all species with a qualitative social process that is guided by respective governance rules. The first validation of the OHSES theoretical framework addressed rabies elimination in Africa, showing that a close cooperation of African states would eliminate rabies in Africa in less than 30 years and result in human capital gains to the gross domestic product of over US$10 billion. A so called Nash equilibrium of best strategies for all actors can be achieved by the inclusion of human capital benefit in the payoff analysis (Bucher *et al*., 2023). The OHSES framework is generic and can address many different communicable and non-communicable health issues related to the sustainable management of natural resources. For example “trade-offs between protecting plant health through use of agrochemicals versus minimizing risks to human health and antimicrobial and insecticide resistance; and ensuring food security by prioritizing the health of crops to maximize agricultural production versus protecting environmental systems critical for human health” (Hoffmann *et al*., 2022). Similar activities take place as part of the Montpellier process (Available at: www.agropolis-fondation.fr/What-is-The-Montpellier-Process-1857, accessed 10 July 2025).

Methods for integrated approaches to health are a rapidly evolving open field, which should aim at qualitative and quantitative studies, valuing both scientific and indigenous knowledge systems on the simultaneous improvement of natural resource management and the health of all species. In Table 1, we restrict to specific techniques and tools and do not repeat the principles of transdisciplinarity and systems thinking. Ultimately, we urgently need more contextualized evidence on the benefits of integrated approaches to health to inform policy and the implementation of programs.

## Discussion

### Current Operationalization of Integrated Approaches To Health

Integrated approaches to health have broad networks of collaborations worldwide. EcoHealth research is conveyed among others through the International Association for Ecology and Health (IAEH) (Available at: www.ecohealthinternational.org, accessed 10 July 2025). The Convention on Biological Diversity (CBD) is an international treaty established during the Earth Summit in Rio de Janeiro in 1992, aiming to conserve biological diversity, ensure its sustainable use, and promote the fair sharing of benefits arising from genetic resources. During the Conference of the Parties 15 (COP15) in December 2022, the CBD adopted the Kunming-Montreal Global Biodiversity Framework (GBF), which underscores the intrinsic link between biodiversity and human health. It advocates for the integration of the One Health approach into National Biodiversity Strategies, which are expected to be the most effective operational tools of EcoHealth and Planetary Health, emphasizing the interconnected health of humans, animals, plants and their shared environment. EcoHealth and Planetary Health recognize that biodiversity is fundamental to human well-being, providing essential services, such as food security, medicinal resources, and clean water. The World Health Organization highlights that biodiversity supports societal needs, including nutrition, energy, and the development of medicines, all of which underpin good health. The GBF sets ambitious targets to halt and reverse biodiversity loss by 2030, recognizing that the degradation of ecosystems directly impacts human health. For instance, the destruction of natural habitats can lead to the emergence of zoonotic diseases, while the loss of medicinal plant species limits potential treatments for various ailments. In urban settings, the integration of green spaces has been shown to reduce non-communicable diseases by promoting physical activity, improving air quality, and reducing stress. The Planetary Health Alliance facilitates international activities (Available at: https://www.planetaryhealthalliance.org/planetary-health, accessed 10 July 2025). The UN Global Climate Action Awards: Planetary Health recognizes and showcase novel solutions by communities, cities, companies, NGOs and other institutions that balance the need for healthy communities with stewardship of natural ecosystems (Available at: https://unfccc.int/climate-action/un-global-climate-action-awards/planetary-health, accessed 10 July 2025).

The four world organizations, the World Health Organization (WHO), the World Organization for Animal Health (WOAH), the Food and Agricultural Organization of the United Nations (FAO) and the United National Environment Programme (UNEP) have agreed to a quadripartite collaboration. As mentioned above, the quadripartite has created a One Health High Level Expert Panel (OHHLEP), which serves as the scientific and strategic advisory group to the Quadripartite organizations. Larger regional bodies, like the G7, have adopted One Health as a guiding principle for societal problem solving on pandemic prevention, antimicrobial resistance management, biodiversity loss and climate change (Anonymous, 2021). Most recently, the scientific advice mechanism (SAM) of the European Union commissioned a report on the operationalization of One Health in the European Union (Group of Chief Scientific Advisors, 2024; SAPEA, 2024). Many countries across the world have already established operational One Health platforms to facilitate multi-sector collaboration in the framework of the International Health Regulations (Zinsstag *et al*., 2023a; Winkler *et al*., 2025) (Table 2).

Among the three integrated approaches, One Health is currently the only one that is operationalized at the level of the international organizations, regional organizations, national governments *and* non-governmental organizations. However, the three integrated concepts should not be seen in competition but contribute to each other mutually because integrated approaches to health are pivotal for complex problem solving such as pandemic prevention, climate change, biodiversity loss and antimicrobial resistance. The leadership of the Quadripartite is important for facilitating global cooperation at the levels of the International Health Regulations (de la Rocque *et al*., 2021) and One Health joint plan of action (2022–2026) (FAO *et al*., 2022) and the forthcoming agreement on a pandemic treaty.

The complex crises that the world is currently facing need integrated approaches, and this article should help to show the strengths and weaknesses of the three best-known approaches in order to help researchers and policy makers to decide how to approach a particular health issue in a particular context. More effective linkages between health and climate action, reduction of biodiversity loss, food security, food systems and antimicrobial resistance, as well as continued strengthening of equity and inclusiveness, will be at the center of attention of integrated approaches to health. Ultimately, national governments are the engines of change, through closer cooperation and communication between institutions and communities at all levels of operational governance.

## Figures and Tables

**Table 1:** Selected key integrated methods and case examples.

Integrated concept	Method	Realm of case study					Case study
		Humans	Domestic animals	Wildlife	Environment	Trans disciplinary	
EcoHealth	Systemic assessment of chemical contamination.	X		X	X	X	Forget and Lebel (2001)
	Action oriented research (Charron, 2012b)	X	X	X	X	X	
	Multi-level interventions	X	X	X	X	X	Lebel (2002)
Planetary health	Health Impact assessment (Osofsky and Pongsiri, 2018)	X			X	X	Winkler *et al*. (2013); Winkler and Utzinger (2014)
	Available (accessed 10 July 2025) at: https://www.planetaryhealthal-liance.org/phcs61820	X			X	X	
	Modeling and scenario analysis of earth and health systems	X			X		Meinshausen *et al*. (2024)
One Health added value	Facilitating joint land-use planning to mitigate conflict at the wildlife/ livestock interface	X	X	X	X	X	Thomson *et al*. (2013)
	Joint human and animal health service delivery (Schelling and Hattendorf, 2015)	X	X			X	Schelling *et al*. (2007a)
	Benefit-cost analysis of zoonoses control (Zinsstag *et al*., 2007)	X	X				Roth *et al*. (2003)
	Benefit-cost analysis of integrated surveillance-response systems (World Bank, 2012)	X	X	X	X		Paternoster *et al*. (2017)
	Equity effectiveness of health interventions (Zinsstag *et al*., 2011a)	X	X	X		X	Mosimann *et al*. (2017)
	Material flow analysis - quantitative microbial risk analysis (MFA- QMRA) (Nguyen-Viet *et al*., 2009)	X	X		X	X	Pham Duc *et al*. (2011)
One Health optimization and balancing	Stabilizing dog populations and improving animal and public health	X	X			X	Schurer *et al*. (2015)
	Integrated AMR surveillance (Zinsstag *et al*., 2011a, 2011b)	X	X		X	X	Abukhattab *et al*. (2023)
	One Health in Social - Ecological Systems (OHSES) (Zinsstag *et al*., 2024)	X	X		X	X	Bucher *et al*. (2023)

**Table 2:** Examples of levels of operationalization of integrated approaches to health.

Integrated concept	Academic networks	International organizations	Regional organizations	National governments	Non-governmental organizations
EcoHealth	E.g. International Organization for Ecology and Health	EcoHealth International			EcoHealth Alliance
Planetary Health	E.g. Planetary Health Alliance	UNDP, WHO, COP		COP	Planetary Health Alliance
One Health	Multiple academic networks and organizations, e.g. AHEAD	Quadripartite OHHLEP	G7, G20, European Union, ECOWAS, African Union, Pacific Community	National One Health platforms, policies and strategic plans	e.g. WCS, WWF

## Data Availability

This work did not include data analysis, hence there is no data available.

## One Health impact statement

In this article, we compare the different paradigms of EcoHealth, One Health and Planetary Health as leading integrated approaches to health. We examine how they are shaping public health and closely related disciplines, how they relate to each other and how they possibly converge (Winkler *et al*., 2025). Compared to public health, integrated approaches to health extend the focus of attention from humans only (anthropocentric perspective) to humans in their socio-cultural and ecological environment, paying attention to inter-species interdependencies. All integrated approaches claim that they are open to epistemic diversity whenever they engage in inter- and transdisciplinary processes, including with indigenous knowledge systems. Integrated approaches to health emphasize systems thinking that recognizes the interconnectedness of ecosystems, human and animal health, and societal factors. They aim to co-create transformational knowledge through participatory transdisciplinary processes between researchers, communities and authorities. Between and within these approaches, variable levels of attention are given to social equity and ecological sustainability.

## References

[R1] Abukhattab S, Hosch S, Abu-Rmeileh NME, Hasan S, Vonaesch P (2023). Whole-genome sequencing for One Health surveillance of antimicrobial resistance in conflict zones: A case study of salmonella spp. and campylobacter spp. in the West Bank, Palestine. Applied and Environmental Microbiology.

[R2] Acheson D, Company PIAL (1988). House of Commons Parliamentary Papers Online.

[R3] Almada AA, Golden CD, Osofsky SA, Myers SS (2017). A case for planetary health/GeoHealth. Geohealth.

[R4] Anonymous (2021). G7 Carbis Bay Health Declaration.

[R5] Assmuth T, Chen X, Degeling C, Haahtela T, Irvine KN (2020). Integrative concepts and practices of health in transdisciplinary social ecology. Social-Ecological Practice Research.

[R6] Azari R, Borisch B (2023). What is public health? A scoping review. Archives of Public Health.

[R7] Baer HA, Singer M, Susser I (2003). Medical Anthropology and the World System.

[R8] Barbour IG (1997). Religion and Science: Historical and Contemporary Issues.

[R9] Brady D (2023). Discussion Paper Social Europe And Well-Being Programme.

[R10] Brown H, Nading AM (2019). Introduction: Human animal health in medical anthropology. Medical Anthropology Quarterly.

[R11] Bucher A, Dimov A, Fink G, Chitnis N, Bonfoh B, Zinsstag J (2023). Benefit-cost analysis of coordinated strategies for control of rabies in Africa. Nature Communications.

[R12] Bunch MJ, Morrison KE, Parkes MW, Venema HD (2011). Promoting health and well-being by managing for social–ecological resilience: The potential of integrating Ecohealth and water resources management approaches. Ecology and Society.

[R13] Charron DF (2012a). Ecosystem approaches to health for a global sustainability agenda. EcoHealth.

[R14] Charron DFE (2012b). Ecohealth Research in Practice.

[R15] Cook RA, Karesh WB, Osofsky SA (2004). The Manhattan Principles on “One World, One Health”: Building Interdisciplinary Bridges to Health in a Globalized World.

[R16] Coreil J (2008). Social Science Contributions to Public Health: Overview International Encyclopedia of Public Health.

[R17] de Castaneda RR, Villers J, Guzman CAF, Eslanloo T, de Paula N (2023). One Health and planetary health research: Leveraging differences to grow together. Lancet Planetary Health.

[R18] de la Rocque S, Belot G, Errecaborde KMM, Sreedharan R, Skrypnyk A (2021). Operationalisation of consensual One Health roadmaps in countries for improved IHR capacities and health security. BMJ Global Health.

[R19] FAO, WHO, and WOAH (2022). One Health Joint Plan of Action (2022–2026) Working Together for the Health of Humans, Animals, Plants and the Environment.

[R20] Forget G, Lebel J (2001). An ecosystem approach to human health. International Journal of Occupational and Environmental Health.

[R21] Gabriel M (2022). Der Mensch Als Tier: Warum Wir Trotzdem Nicht in Die Natur Passen.

[R22] Mechanism SA, Group of Chief Scientific Advisors (2024). One Health governance in the European Union. Scientific Opinion European Comission in Brussels, Belgium.

[R23] Guimarães JRD, Mergler D, Charron DE (2012). Ecohealth Research in Practice Insight and Innovation in International Development.

[R24] Guthrie AG, Taylor WW, Muir AM, Frank KA, Regier HA (2019). The role of a multi-jurisdictional organization in developing ecosystem-based management for fisheries in the Great Lakes basin. Aquatic Ecosystem Health and Management.

[R25] Haraway DJ (2016). Staying with the Trouble: Making Kin in the Chtulucene.

[R26] Hardin G (1968). The tragedy of the commons. The population problem has no technical solution; it requires a fundamental extension in morality. Science.

[R27] Harrison S, Kivuti-Bitok L, Macmillan A, Priest P (2019). EcoHealth and One Health: A theory-focused review in response to calls for convergence. Environment International.

[R28] Herweg K, Schäfer N, Zimmermann A (2012). Guidelines for Integrative Training in Inter- and Transdisciplinary Research Settings Hints and Tools for Trainers of Trainers.

[R29] Hillier SA, Taleb A, Chaccour E, Aenishaenslin C (2021). Examining the concept of One Health for indigenous communities: A systematic review. One Health.

[R30] Hoffmann V, Paul B, Falade T, Moodley A, Ramankutty N (2022). A One Health approach to plant health. CABI Agriculture and Bioscience.

[R31] Holling CS (2001). Understanding the complexity of economic, ecological, and social systems. Ecosystems.

[R32] Hoogeveen D, Atleo CG, Patrick L, Kennedy AM, Leduc M (2023). On the possibility of decolonising planetary health: Exploring new geographies for collaboration. Lancet Planetary Health.

[R33] Johnson KL, Gislason MK, Silva DS, Smith MJ, Buse C (2024). Advancing planetary health through interspecies justice: A rapid review. Challenges.

[R34] Johnston FH, Jacups SP, Vickery AJ, Bowman D (2013). Ecohealth and aboriginal health: A review of common ground. EcoHealth.

[R35] Kickbusch I, Bright RA (2024). Governing global health with a planetary mindset. BMJ.

[R36] Kirksey SE, Helmreich S (2010). The emergence of multispecies ethnography. Cultural Anthropology.

[R37] Lebel J (2002). Health: An Ecosytem Approach.

[R38] Lerner H, Berg C (2017). A comparison of three holistic approaches to health: One Health, EcoHealth, and planetary health. Frontiers in Veterinary Science.

[R39] Lovelock JE (1972). Gaia as seen through the atmosphere. Atmoshperic Environment.

[R40] Malsch AKF, Killin A, Kaiser MI (2024). Health-oriented environmental categories, individual health environments, and the concept of environment in public health. Health Care Analysis.

[R41] McGinnis MD, Ostrom E (2014). Social-ecological system framework: Initial changes and continuing challenges. Ecology and Society.

[R42] McMichael AJ (2013). Globalization, climate change, and human health. The New England Journal of Medicine.

[R43] Meinshausen M, Schleussner CF, Beyer K, Bodeker G, Boucher O (2024). A perspective on the next generation of earth system model scenarios: Towards representative emission pathways (REPs). Geoscientific Model Development.

[R44] Moon K, Blackman D (2014). A guide to understanding social science research for natural scientists. Conservation Biology.

[R45] Mosimann L, Traore A, Mauti S, Lechenne M, Obrist B (2017). A mixed methods approach to assess animal vaccination programmes: The case of rabies control in Bamako, Mali. Acta Tropica.

[R46] Myers SS, Gaffikin L, Golden CD, Ostfeld RS, Redford KH (2013). Human health impacts of ecosystem alteration. Proceedings of the National Academy of Sciences of the United States of America.

[R47] Nguyen-Viet H, Zinsstag J, Schertenleib R, Zurbrugg C, Obrist B (2009). Improving environmental sanitation, health, and well-being: A conceptual framework for integral interventions. EcoHealth.

[R48] Nowak MA, Coakley S (2013). Evolution, Games and God: The Principle of Cooperation.

[R49] Obrist B, Iteba N, Lengeler C, Makemba A, Mshana C (2007). Access to health care in contexts of livelihood insecurity: A framework for analysis and action. PLoS Medicine.

[R50] Obrist B, Pfeiffer C, Henley R (2010). Multi-layered social resilience: A new approach in mitigation research. Progress in Development Studies.

[R51] Adisasmito WB, Almuhairi S, Behravesh CB, Bilivogui P, One Health High-Level Expert (2022). One Health: A new definition for a sustainable and healthy future. PLoS Pathogens.

[R52] Osofsky SA, Pongsiri MJ (2018). Operationalising planetary health as a game-changing paradigm: Health impact assessments are key. Lancet Planetary Health.

[R53] Osofsky SA, Cleaveland S, Karesh WB, Kock MD, Nyhus PJ, Starr L, Yang A (2005). Conservation and Development Interventions at the Wildlife/Livestock Interface: Implications for Wildlife.

[R54] Ostrom E (2015). Governing the Commons : The Evolution of Institutions for Collective Action.

[R55] Parkes MW, Stephen C (2020). Animals, Health, and Society.

[R56] Paternoster G, Babo Martins S, Mattivi A, Cagarelli R, Angelini P (2017). Economics of One Health: Costs and benefits of integrated West Nile virus surveillance in Emilia-Romagna. PLoS One.

[R57] Pelluchon C (2021). Das Zeitalter Des Lebendigen: Eine Neue Philosophie der Aufklärung.

[R58] Pettan-Brewer C, Penn G, Biondo AW, Jaenisch T, Grutzmacher K, Kahn LH (2024). Who coined the term “One Health”? Cooperation amid the siloization. One Health.

[R59] Duc Pham, Nguyen-Viet H, Hattendorf J, Zinsstag J, Dac Cam P, Odermatt P (2011). Risk factors for Entamoeba histolytica infection in an agricultural community in Hanam province, Vietnam. Parasites and Vectors.

[R60] Rapport D, Costanza R, Epstein PR, Gaudet C, Levins R (1998). Ecosystem Health.

[R61] Rapport D, Böhm G, Buckingham D, Cairns J, Costanza R (1999). Ecosystem health: The concept, the ISEH, and the important tasks ahead. Ecosystem Health.

[R62] Rock M, Buntain BJ, Hatfield JM, Hallgrimsson B (2009). Animal-human connections, “One Health,” and the syndemic approach to prevention. Social Science and Medicine.

[R63] Roth F, Zinsstag J, Orkhon D, Chimed-Ochir G, Hutton G (2003). Human health benefits from livestock vaccination for brucellosis: Case study. Bulletin of the World Health Organization.

[R64] Rüegg S, Häsler B, Zinsstag J (2018). Integrated Approaches to Health: A Handbook for the Evaluation of One Health.

[R65] Santos O, Virgolino A, Santos RR, Costa J, Rodrigues A, Vaz-Carneiro A (2019). Environmental Health: An Overview on the Evolution of the Concept and its Definitions, Enyclopedia of Environmental Health.

[R66] SAPEA (2024). One Health governance in the European Union.

[R67] Schelling E, Hattendorf J (2015). One Health study designs. One Health: The Theory and Practice of Integrated Health Approaches.

[R68] Schelling E, Bechir M, Ahmed MA, Wyss K, Randolph TF, Zinsstag J (2007a). Human and animal vaccination delivery to remote nomadic families, Chad. Emerging Infectious Diseases.

[R69] Schelling E, Wyss K, Diguimbaye C, Bechir M, Taleb MO, Hirsch Hadorn G, Hoffmann-Reim H, Biber-Klemm S, Grossenbacher W, Joye D, Pohl C, Wiesmann U, Zemp E (2007b). Handbook of Transdisciplinary Research A Proposition by the Swiss Academies of Arts and Sciences.

[R70] Schurer JM, Phipps K, Okemow C, Beatch H, Jenkins E (2015). Stabilizing dog populations and improving animal and public health through a participatory approach in indigenous communities. Zoonoses and Public Health.

[R71] Schwabe CW (1984). Veterinary Medicine and Human Health.

[R72] Singer M, Clair S (2003). Syndemics and public health: Reconceptualizing disease in bio-social context. Medical Anthropology Quarterly.

[R73] Steele JH (1949). Veterinary public health. The North American Veterinarian.

[R74] Thomson GR, Penrith ML, Atkinson MW, Atkinson SJ, Cassidy D, Osofsky SA (2013). Balancing livestock production and wildlife conservation in and around southern Africa’s transfrontier conservation areas. Transboundary and Emerging Diseases.

[R75] Waltner-Toews D (2001). An ecosystem approach to health and its applications to tropical and emerging diseases. Cadernos de Saúde Pública.

[R76] Webb JC, Mergler D, Parkes MW, Saint-Charles J, Spiegel J (2010). Tools for thoughtful action: The role of ecosystem approaches to health in enhancing public health. Canadian Journal of Public Health Revue Canadienne de Sante Publique.

[R77] Webb J, Raez-Villanueva S, Carriere PD, Beauchamp AA, Bell I (2023). Transformative learning for a sustainable and healthy future through ecosystem approaches to health: Insights from 15 years of co-designed ecohealth teaching and learning experiences. Lancet Planetary Health.

[R78] Weiss MG (2001). Cultural epidemiology : An introduction and overview. Anthropology & Medicine.

[R79] Whitmee S, Haines A, Beyrer C, Boltz F, Capon AG (2015). Safeguarding human health in the Anthropocene epoch: Report of the Rockefeller Foundation-lancet commission on planetary health. Lancet.

[R80] Whittaker M, Zinsstag J, Schelling E, Waltner-Toews D, Whittaker M, Tanner M (2015). One Health: The Theory and Practice of Integrated Approaches to Health.

[R81] Whittaker M, Obrist B, Berger-Gonzalez M, Zinsstag J, Schelling E, Crump L, David Whittaker M, Tanner M, Stephen C (2020). One Health: The Theory and Practice of Integrated Health Approaches.

[R82] Wilcox BA, Aquirre AA (2004). One ocean, one health. EcoHealth.

[R83] Winkler MS, Utzinger J (2014). The search for underlying principles of health impact assessment: Progress and prospects: Comment on “investigating underlying principles to guide health impact assessment”. International Journal of Health Policy and Management.

[R84] Winkler MS, Krieger GR, Divall MJ, Cisse G, Wielga M (2013). Untapped potential of health impact assessment. Bulletin of the World Health Organization.

[R85] Winkler AS, Brux CM, Carabin H, Neves das, Hasler B (2025). The lancet One Health commission: Harnessing our interconnectedness for equitable, sustainable, and healthy socioecological systems. Lancet.

[R86] Woods A, Bresalier M, Cassidy A, Mason Dentinger R (2017). Animals and the Shaping of Modern Medicine: One Health and its Histories.

[R87] World Bank (2012). People, Pathogens and Our Planet: Volume 2: The Economics of One Health, Report No. 69145-GLB.

[R88] World Commission on the Environment and Development (1987). Our Common Future.

[R89] Zinsstag J (2012). Convergence of EcoHealth and One Health. EcoHealth.

[R90] Zinsstag J, Schelling E, Wyss K, Bechir M (2005). Potential of cooperation between human and animal health to strengthen health systems. Lancet.

[R91] Zinsstag J, Taleb Ould, Craig PS (2006). Editorial: Health of nomadic pastoralists: New approaches towards equity effectiveness. Tropical Medicine and International Health.

[R92] Zinsstag J, Schelling E, Roth F, Bonfoh B, de Savigny D, Tanner M (2007). Human benefits of animal interventions for zoonosis control. Emerging Infectious Diseases.

[R93] Zinsstag J, Dürr S, Penny MA, Mindekem R, Roth F (2009). Transmission dynamics and economics of rabies control in dogs and humans in an African city. Proceedings of the National Academy of Sciences of the United States of America.

[R94] Zinsstag J, Schelling E, Waltner-Toews D, Tanner M (2011a). From “One Medicine” to “One Health” and systemic approaches to health and well-being. Preventive Veterinary Medicine.

[R95] Zinsstag JB, Cissé G, Nguyen-Viet H, Silué B, Nguessan TS (2011b). Perspectives of the Swiss National Centre of Competence in Research (NCCR).

[R96] Zinsstag J, Meisser A, Schelling E, Bonfoh B, Tanner M (2012). From ‘Two medicines’ to ‘One Health’ and beyond. The Onderstepoort Journal of Veterinary Research.

[R97] Zinsstag J, Mahamat Bechir, Schelling E, Zinsstag J, Schelling E, Crump L, Whittaker M, Tanner M, Stephen C (2020). One Health: The Theory and Practice of Integrated Health Approaches.

[R98] Zinsstag J, Hediger K, Osman YM, Abukhattab S, Crump L (2022). The promotion and development of One Health at Swiss TPH and its greater potential. Diseases.

[R99] Zinsstag J, Kaiser-Grolimund A, Heitz-Tokpa K, Sreedharan R, Lubroth J (2023a). Advancing one human-environmental-animal health for Global Health security: What does the evidence say?. Lancet.

[R100] Zinsstag J, Pelikan K, Gonzalez Berger, Kaiser-Grolimund A, Crump L, Lawrence RJ (2023b). Handbook of Transdisciplinarity: Global Perspective.

[R101] Zinsstag J, Meyer JM, Bonfoh B, Fink G, Dimov A (2024). One Health in human-environment systems: Linking health and the sustainable use of natural resources. CABI One Health.

